# Does rumination mediate the relationship between emotion regulation ability and posttraumatic stress disorder?

**DOI:** 10.3402/ejpt.v5.23547

**Published:** 2014-08-26

**Authors:** Thomas Ehring, Anke Ehlers

**Affiliations:** 1 Department of Clinical Psychology, Institute of Psychology, University of Münster, Münster, Germany; 2 Department of Experimental Psychology, University of Oxford, Oxford, UK

**Keywords:** Trauma, PTSD, rumination, emotional regulation

## Abstract

**Background and objectives:**

Trauma-related rumination has been suggested to be involved in the maintenance of posttraumatic stress disorder (PTSD). This view has empirically been supported by extensive evidence using cross-sectional, prospective, and experimental designs. However, it is unclear why trauma survivors engage in rumination despite its negative consequences. The current study aimed to explore the hypothesis that low emotion regulation ability underlies trauma-related rumination.

**Methods:**

Emotion regulation ability and trauma-related rumination were assessed in 93 road traffic accident survivors 2 weeks post-trauma. In addition, symptom levels of PTSD were assessed at 2 weeks as well as 1, 3, and 6 months follow-up.

**Results:**

Emotion regulation ability was significantly related to trauma-related rumination as well as levels of PTSD symptoms. In addition, the association between low emotion regulation ability and PTSD was mediated by rumination.

**Conclusions:**

The findings support the view that rumination is used as a dysfunctional emotion regulation strategy by trauma survivors.

Many trauma survivors experience some degree of posttraumatic stress symptoms shortly after the traumatic experience (McFarlane, 2000). Whereas most survivors show spontaneous recovery from these symptoms, a substantial minority develops chronic posttraumatic stress disorder (PTSD). This raises the important question about which processes are involved in the maintenance of PTSD symptoms in the latter group.

Trauma-related rumination, defined as repetitive and recurrent thinking about the trauma and its consequences (Michael, Halligan, Clark, & Ehlers, 2007), has been suggested to be a dysfunctional cognitive coping strategy that maintains PTSD symptoms (Ehlers & Clark, 2000; Wells & Sembi, 2004). There is accumulating evidence that trauma-related rumination is phenomenologically and functionally different from intrusive memories in PTSD. Intrusive memories are predominantly sensory experiences of short duration that represent the experience of the trauma itself, whereas rumination is predominantly described as a train of thoughts of longer duration that elaborate on the experience (e.g., Evans, Ehlers, Mezey, & Clark, 2007; Speckens, Ehlers, Hackmann, Ruths, & Clark, 2007). This distinction between rumination and re-experiencing is now also explicitly mentioned in DSM-5's description of the diagnostic features of PTSD (APA, 2013).

In line with the view of trauma-related rumination as a maintaining factor, there is evidence from cross-sectional studies showing significant and substantial correlations between trauma-related rumination and either symptom levels or a diagnosis of PTSD following different types of traumatic events, including road traffic accidents (e.g., Ehlers, Mayou, & Bryant, 1998; Ehring, Frank, & Ehlers, 2008), assault (e.g., Dunmore, Clark, & Ehlers, 1999; Michael et al., 2007), and work-related trauma in emergency personnel (e.g., Clohessy & Ehlers, 1999; Razik, Ehring, & Emmelkamp, 2013). Importantly, there is additional evidence suggesting that rumination is not just an epiphenomenon or a consequence of having the disorder, but may play a causal role in the maintenance of PTSD. Results from a series of prospective studies show that trauma-related rumination assessed shortly after the trauma predicts PTSD some months later, even after controlling for initial symptom severities and/or other known predictors of PTSD (e.g., Ehring, Frank, et al., 2008; Kleim, Ehlers, & Glucksman, 2007; Michael et al., 2007). In addition, a series of experimental studies showed that the induction of rumination about a traumatic film led to significantly more analogue PTSD symptoms and/or a significantly slower recovery from the film than different control conditions (e.g., Wells & Papageorgiou, 1995; Zetsche, Ehring, & Ehlers, 2009). The same results were found when inducing rumination about a real-life distressing life event (Ehring, Fuchs, & Kläsener, 2009). In sum, there is converging evidence from cross-sectional, prospective, and experimental studies supporting the view that rumination is involved in the maintenance of PTSD.

One question that has only rarely been addressed to date is why trauma survivors engage in trauma-related rumination despite the negative effects this strategy appears to have. Results from research into repetitive negative thinking in the context of other emotional disorders suggest that rumination may be related to deficits in emotion regulation. For example, depressive rumination has been conceptualized as a dysfunctional emotion regulation strategy (Nolen-Hoeksema, Wisco, & Lyubomirsky, 2008). In addition, there is evidence suggesting that rumination is related to high levels of experiential avoidance and fear of emotions (e.g., Cribb, Moulds, & Carter, 2006; Giorgio et al., 2010). Similarly, there is emerging evidence of an emotion dysregulation model of worry, a process that is closely related to rumination (see Ehring & Watkins, 2008). Specifically, excessive levels of worry and/or a diagnosis of generalized anxiety disorder have been found to be related to deficits in emotion regulation, including poor understanding of emotion, emotional avoidance, and the lack of access to functional emotion regulation strategies (e.g., Mennin, Heimberg, Turk, & Fresco, 2005; Mennin, Holaway, Fresco, Moore, & Heimberg, 2007; Salters-Pedneault, Roemer, Tull, Rucker, & Mennin, 2006).

Rumination and other forms of repetitive negative thinking (e.g., worry) have not only been found to be phenomenologically similar, but also appear to be driven by similar processes across disorders (for a review, see Ehring & Watkins, 2008). It can therefore be hypothesized that deficits in adaptive emotion regulation not only underlie depressive rumination and worry in GAD but may also contribute to PTSD sufferers engaging in trauma-related rumination. In other words, trauma survivors are thought to use trauma-related rumination as a dysfunctional emotion regulation strategy because they lack the ability to regulate their emotions in a more adaptive way. To our knowledge, this idea has not directly been tested to date. However, indirect evidence comes from studies showing associations between PTSD and emotion functioning. Specifically, PTSD has been found to be related to high levels of alexithymia, that is, an inability to experience, identify, and express negative emotions (Frewen, Dozois, Neufeld, & Lanius, 2008), heightened levels of experiential avoidance, fear of emotions and lack of emotional acceptance (e.g., Kashdan, Morina, & Priebe, 2009; Tull, Jakupcak, McFadden, & Roemer, 2007), self-reported difficulties in emotion regulation (Tull, Barrett, McMillan, & Roemer, 2007; van der Kolk, Roth, Pelcovitz, Sunday, & Spinazzola, 2005), and a frequent use of dysfunctional emotion regulation strategies (e.g., emotion suppression) as well as an infrequent use of functional strategies (e.g., cognitive reappraisal) (e.g., Ehring & Quack, 2010; Moore, Zoellner, & Mollenholt, 2008). In sum, past research has shown associations between emotion regulation and PTSD on the one hand and trauma-related rumination and PTSD on the other hand. Theories and findings on other types of repetitive negative thinking suggest a specific relationship between these two predictors of PTSD in that low emotion regulation ability leads to trauma-related rumination, which in turn contributes to the maintenance of PTSD. In other words, the association between low emotion regulation ability and PTSD is suggested to be partially mediated by engagement in trauma-related rumination.

Emotion regulation is a broad concept that has been defined and operationalized in very different ways. However, most conceptualizations agree on a number of key processes, including awareness and understanding of one's emotions, acceptance of emotions, and the ability to successfully use situationally appropriate strategies to regulate one's emotions (e.g., Brackett et al., 2013; Gratz & Roemer, 2004; Gross & Thompson, 2007; Kring & Werner, 2004). Rumination is typically regarded as a dysfunctional emotion regulation strategy in which individuals engage in an inflexible manner because of a reduced ability to successfully regulate negative emotions by other means (Nolen-Hoeksema et al., 2008). Therefore, the current study focused on the ability to manage one's emotions in an adaptive way as a specific aspect of the broader concept of emotion regulation.

The aim of the current study was to test the hypothesis that low emotion regulation ability is one of the reasons why trauma survivors engage in trauma-related rumination. Specifically, we expected that emotion regulation ability would be significantly negatively related to PTSD (Hypothesis 1), emotion regulation ability would be significantly negatively related to levels of trauma-related rumination (Hypothesis 2), and that rumination would mediate the relationship between emotion regulation ability and PTSD (Hypothesis 3).

## Method

### Participants

Participants were drawn from a sample of 147 road traffic accident survivors who were treated for their injuries at the emergency department of a metropolitan hospital. The current article reports on 93 participants who completed the Mayer-Salovey-Caruso Emotional Intelligence Test (MSCEIT) (66.7% male; 75.3% Caucasian; age: *M*=34.98, *SD*=8.86; ethnicity: 68.7% Caucasian). The mean Injury Severity Score in this sample was 2.37 (*SD=*2.64, *min*=0, *max*=13). Inclusion criteria were: Injury in a road traffic accident as a driver, passenger, motorcyclist, or cyclist; injuries more severe than triage category “blue” (very mild injuries); age between 18 and 65; address in local catchment area. Exclusion criteria were: left before receiving medical treatment, attended the emergency department more than 12 hours after the accident, currently psychotic or suicidal, and command of English insufficient to complete measures. Additional information about the sample and the recruitment is given elsewhere (Ehring, Ehlers, & Glucksman, 2008).

### Instruments

#### Emotion regulation ability

The emotion regulation subscale of the MSCEIT (Mayer, Salovey, & Caruso, 2002), an ability measure of emotional intelligence, was used to assess emotion regulation ability. There is extensive research supporting the reliability and validity of the MSCEIT. For example, internal consistencies of *α*=0.77–0.88 have been reported for the different subscales (Mayer, Salovey, Caruso, & Sitarenios, 2003). High correlations with other emotion regulation ability measures and with external criterion variables (e.g., enhanced social relationships with friends or at work) support the validity of the measure (Mayer, Salovey, & Caruso, 2012). The emotion regulation subscale consists of two tasks assessing intrapersonal emotion regulation (20 items) and interpersonal emotion regulation (9 items). In addition to a total emotion regulation score, separate subscores for intrapersonal vs. interpersonal regulation can be computed. Participants are provided with short descriptions of emotion-evoking situations and are asked to rate the effectiveness of different strategies for regulating one's own feelings or managing emotionally challenging interpersonal situations. The MSCEIT scores were computed using the expert criterion, that is, they reflect the degree to which a person's responses match those provided by experts in emotion regulation during the process of validation (see Mayer et al., 2003). In the current study, the hypotheses were tested using the total emotion regulation score. The intrapersonal and interpersonal regulation subscores were included in the correlational analyses in an exploratory way.

#### Trauma-related rumination

The rumination subscale of the *Responses to Intrusions Questionnaire* (RIQ) was used to assess the degree of rumination about the trauma and/or its consequences. The RIQ was developed in a series of studies and trauma survivors’ responses to intrusive memories; it has been shown to possess good reliability and validity, including internal consistencies of *α*=0.80–0.86 and significant and substantial correlations (*r*>0.57) with other rumination measures (Ehring, Frank, et al., 2008; Murray, Ehlers, & Mayou, 2002; Steil & Ehlers, 2000). The rumination subscale comprises eight items (e.g., *I think about why the event happened to me; I dwell on what I should have done differently; I dwell on how the trauma could have been prevented*) that are rated on a scale from 0 (*never*) to 3 (*always*).

#### PTSD symptom severity

The *Posttraumatic Diagnostic Scale* (PDS) (Foa, Cashman, Jaycox, & Perry, 1997) was used to assess symptom levels of PTSD. The PDS is a well-validated and widely used self-report measure of PTSD symptom severity. It shows high internal consistencies (*α*>0.85), and high associations with other symptom measures of PTSD (Foa et al., 1997). The main part of the measure consists of 17 items representing the DSM-IV criteria for PTSD. Each item is rated on a scale from 0 (*not at all or only one time*) to 3 (*five or more times a week/almost always*).

### Procedure

At 2 weeks post-trauma, participants attended a research session, in which participants completed the MSCEIT, RIQ and PDS. It also contained some additional tasks and questionnaires unrelated to the analyses presented here (see Ehring, Ehlers, et al., 2008). At 1, 3, and 6 months follow-up, participants completed the PDS again. The study was approved by the local Research Ethics Committees. At the first assessment, participants provided informed written consent. At the end of the study, participants received a reimbursement of £50.

### Statistical analyses

PDS scores were skewed. Therefore, Spearman's rank correlation coefficients (Spearman's *rho*) were computed to assess associations between the different variables of interest. In order to test the mediation model, a nonparametric resampling approach (bootstrapping) suggested by Preacher and Hayes (2008) was used.

## Results

Table 1 shows Spearman's rank correlations between all variables of interest.

**Table 1:** Correlations between variables of interest (Spearman's Rho)

	MSCEIT-ER	MSCEIT-intra	MSCEIT-inter	RIQrum	PDS 2w	PDS 1m	PDS 3m	PDS 6m
MSCEIT-ER	–	0.89***	0.88***	−0.25*	−0.21*	−0.17	−0.28*	−0.27*
MSCEIT-intra		–	0.58***	−0.21*	−0.25*	−0.21+	−0.31**	−0.25*
MSCEIT-inter			–	−0.22*	−0.13	−0.09	−0.16	−0.23*
RIQrum				–	0.53***	0.56***	0.52***	0.53***
PDS 2w					–	0.83***	0.67***	0.59***
PDS 1m						–	0.84***	0.68***
PDS 3m							–	0.71***

MSCEIT-ER=MSCEIT emotion regulation score; MSCEIT-intra=MSCEIT intrapersonal emotion regulation subscore; MSCEIT-inter=MSCEIT interpersonal emotion regulation subscore; RIQrum=Responses to Intrusions Questionnaire—rumination subscale; PDS=Posttraumatic Diagnostic Scale; 2w=2 weeks; 1m=1 month; 3m=3 months; 6m=6 months.

*** *p<*0.001;

** *p*<0.01;

* *p*<0.05;

+ *p*<0.10.

### Hypothesis 1: Relationship between emotion regulation ability and PTSD

As hypothesized, the MSCEIT emotion regulation score showed a significant negative correlation with PTSD symptom severities at 2 weeks, 3 and 6 months post-trauma. However, no significant correlation emerged for symptom scores at 1-month post-trauma. Parallel results were found for the MSCEIT *intra*personal emotion regulation subscore. However, the *inter*personal emotion regulation subscore was only significantly correlated with PTSD symptoms at 6 months follow-up.

### Hypothesis 2: Relationship between emotion regulation ability and rumination

In line with the second hypothesis, significant negative correlations between trauma-related rumination and all three MSCEIT scores were found.

### Hypothesis 3: Mediation

Our third hypothesis was that the relationship between emotion regulation ability and PTSD would be mediated by trauma-related rumination. To test this hypothesis, three nonparametric mediation analyses using bootstrapping were conducted. In all three analyses, the total MSCEIT emotion regulation score was the independent variable and the RIQ rumination score was the mediator. Dependent variables were PTSD symptom severity scores at 2 weeks, 3 and 6 months, respectively.^1^ As shown in Table 2, the indirect effects in all analyses were significant, whereas the direct effect (c’) was not significant in any of these analyses.

**Table 2:** Results of mediation analyses

			Effect of IV on M (a)		Effect of M on DV (b)		Direct effect (c’)		Indirect effect (a×b)		
						
Independent variable (IV)	Mediator (M)	Dependent variable (DV)	*B*	*SE(B)*	*B*	*SE(B)*	*B*	*SE(B)*	*B*	*SE(B)*	95% CI
MSCEIT-ER	RIQ rum	PDS 2w	−0.11**	0.04	1.72***	0.22	0.02	0.08	−0.18**	0.07	[−0.33; −0.06]
MSCEIT-ER	RIQ rum	PDS 3m	−0.09*	0.03	1.74***	0.22	−0.001	0.08	−0.16*	0.07	[−0.30; −0.05]
MSCEIT-ER	RIQ rum	PDS 6m	−0.11**	0.04	0.93***	0.19	−0.05	0.07	−0.10*	0.04	[−0.19; −0.04]
RIQ rum	MSCEIT-ER	PDS 2w	−0.87**	0.29	0.02	0.08	1.72***	0.22	−0.02	0.08	[−0.22; 0.11]
RIQ rum	MSCEIT-ER	PDS 3m	−0.84**	0.29	−0.09	0.07	1.47***	0.21	0.08	0.09	[−0.05; 0.32]
RIQ rum	MSCEIT-ER	PDS 6m	−0.88**	0.29	−0.05	0.07	0.93***	0.19	0.05	0.08	[−0.10; 0.22]

Path/regression coefficients are unstandardized.

PDS=Posttraumatic Diagnostic Scale; 2w=2 weeks; 1m=1 month; 3m=3 months; 6m=6 months; RIQ rum=Responses to Intrusions Questionnaire—rumination subscale.

* *p<*0.05;

** *p*<0.01;

*** *p*<0.001.

In order to test the specificity of the direction of our effects, we additionally ran three analyses testing the reverse mediation with the RIQ score (rumination) as the independent variable and the emotion regulation ability score (MSCEIT) as the mediator. As shown in Table 2, the indirect effects were non-significant in all three analyses, with a significant direct effect of rumination on PTSD.

## Discussion

All three hypotheses were supported by the data. In line with the first hypothesis, low emotion regulation ability was significantly related with PTSD symptom severity concurrently (at 2 weeks post-trauma) as well as prospectively (3 and 6 months post-trauma). This replicates earlier findings showing an association between emotion regulation difficulties and PTSD in trauma survivors (e.g., Ehring & Quack, 2010; Moore et al., 2008; Tull, Barrett, et al., 2007). Whereas earlier research was exclusively based on self-report, the current study used an ability measure of emotion regulation. Scores on the ability measure can be expected to be less influenced by current symptom severities than self-report measures traditionally used in the field (Mayer, Caruso, & Salovey, 2000). When looking at the two MSCEIT subscales, results were more consistent for the *intra*personal emotion regulation score than for the score representing *inter*personal emotion regulation. This is not surprising as managing negative intrapersonal experiences (intrusive memories, negative emotions) can be seen as the main challenge for trauma survivors.

As predicted, there was also a significant negative association between low emotion regulation ability and the degree to which participants reported engaging in trauma-related rumination. Importantly, results of the mediation analyses showed that the effect of low emotion regulation ability on PTSD symptoms was mediated by rumination. This was not only true for levels of PTSD assessed concurrently, but also for the prospective association between emotion regulation ability and PTSD up to 6 months later. The direction of this mediation effect was specific as emotion regulation ability did not mediate the association between rumination and PTSD in an additional set of analyses.

The pattern of findings is in line with the view that difficulties regulating negative emotions following trauma may prompt trauma survivors to engage in trauma-related rumination to cope with their experience. It parallels similar findings on other types of repetitive negative thinking, such as depressive rumination (Nolen-Hoeksema et al., 2008) and GAD-type worry (Mennin et al., 2005). The results can also be seen as supporting a transdiagnostic account of repetitive negative thinking, with the key assumption that rumination and worry are not only phenomenologically similar but also driven by parallel processes across disorders (Ehring & Watkins, 2008).

To our knowledge, this is the first study investigating the relationship of emotion regulation and rumination in predicting PTSD. Individual differences in the ability to regulate emotions contributed to trauma-related rumination and may add to other processes such as unhelpful appraisals that motivate rumination (Kleim, Ehlers, & Glucksman, 2012). The study has a number of strengths, including the use of an ability measure of emotion regulation, a relatively large sample size and a prospective design. On the other hand, some limitations are noteworthy. First, only road traffic accident survivors were included in the current study. Future research should test whether results generalize to PTSD following other types of traumatic events. Second, as described in the introduction emotion regulation is a broad concept that includes many different processes. Although the emotion regulation abilities assessed here are arguably the most relevant one for the purpose of our study, future research should nevertheless aim at assessing emotion regulation in a broader way in order to test which aspects of emotion regulation are most closely related to rumination. Assessment tools could include measures assessing emotion regulation ability in a multidimensional way (e.g., Difficulties in Emotion Regulation Scale; Gratz & Roemer, 2004), as well as instruments assessing typical emotion regulation strategy use (e.g., Emotion Regulation Questionnaire; Gross & John, 2003). Finally, emotion regulation ability and rumination were assessed at the same time. In order to demonstrate that emotion regulation difficulties are indeed an antecedent of rumination a different design is needed where both variables are assessed repeatedly over time. Ideally, such a prospective study should also include a pre-trauma assessment; however, it should be noted that such a design is extremely challenging for practical reasons.

Despite these limitations, the current study provides evidence for the view that rumination is at least partly due to trauma survivors’ difficulties in regulating emotions. If replicated in future research, this may also have clinical implications. First, there is preliminary evidence that high levels of trauma-related rumination interfere with successful treatment of PTSD (Echiverri, Jaeger, Chen, Moore, & Zoellner, 2011), which may suggest the necessity to directly target rumination in PTSD treatment. When developing interventions targeting trauma-related rumination, an emotion regulation framework may prove fruitful (see Mennin, 2004, for a similar approach in the treatment of GAD-type worry). Second, as rumination has been identified as a stable predictor of PTSD following trauma targeting this process may also be helpful for the prevention of the disorder (see also Topper, Emmelkamp, & Ehring, 2010). The findings of the current study suggest that the promotion of adaptive emotion regulation strategies may be a promising strategy in this context.

However, it should also be noted that the magnitude of the effects involving the emotion regulation ability measure was only modest, which limits the conclusions that can be drawn regarding the clinical relevance of the current findings. Most importantly, the current study therefore points towards the necessity to further investigate the processes that can explain why trauma survivors engage in trauma-related rumination despite its proven negative consequences.

## Footnotes

1 No mediation analysis was conducted with PDS scores at 1 month as no significant associations between MSCEIT and PDS were found at this time point (see Table 1).
